# Gesundheitliche Versorgung von Menschen ohne gültige Aufenthaltspapiere in Deutschland – Ein Scoping-Review

**DOI:** 10.1007/s00103-026-04210-0

**Published:** 2026-03-12

**Authors:** Carolin Ochs, Maike Grube, Anja Dieterich

**Affiliations:** 1 Berlin School of Public Health, Alice Salomon Hochschule Berlin, Berlin, Deutschland; 2 Diakonie Deutschland, Evangelisches Werk für Diakonie und Entwicklung e. V., Caroline-Michaelis-Str. 1, 10115 Berlin, Deutschland; 3 Alice Salomon Hochschule Berlin, Berlin, Deutschland; 4 Fachbereich 2: Gesundheit, Erziehung und Bildung, Alice Salomon Hochschule Berlin, Berlin, Deutschland

**Keywords:** Undokumentierte Migrant:innen, Menschen ohne Papiere, Menschenrecht auf Gesundheit, Zugangsbarrieren, Nichtversicherte, Undocumented migrants, Undocumented people, Human right to health, Access barriers, Uninsured

## Abstract

**Hintergrund:**

Menschen ohne gültige Aufenthaltspapiere haben in Deutschland einen erschwerten Zugang zur Gesundheitsversorgung. Sie haben Anspruch auf eingeschränkte Gesundheitsleistungen nach dem Asylbewerberleistungsgesetz, können diesen aufgrund der Übermittlungspflichten der Sozialbehörden jedoch nicht einlösen, ohne Aufdeckung und Abschiebung zu riskieren. Mit der vorliegenden Arbeit wird der Forschungsstand zur gesundheitlichen Versorgung dieser Bevölkerungsgruppe erstmals systematisch aufgearbeitet.

**Methoden:**

Die Literaturrecherche erfolgte nach dem PRISMA Statement für Scoing Reviews (PRISMA-ScR) in den Datenbanken Medline, CINAHL, PSYNDEX, SocINDEX und Juris sowie mittels ergänzender E‑Mail-Anfragen und per Handsuche. Eingeschlossen wurden Arbeiten aus den Jahren 2005–2024. Die Daten wurden im Hinblick auf rechtliche, strukturelle und praktische Zugangsbarrieren und Versorgungslücken sowie auf empfohlene Maßnahmen analysiert.

**Ergebnisse:**

Es konnten insgesamt 88 Arbeiten eingeschlossen werden, die mehrheitlich auf quantitativen Datenerhebungen basieren. Die vorliegenden Studien zeigen, dass der Zugang zu gesundheitlicher Versorgung für Menschen ohne gültige Aufenthaltspapiere zumeist nicht über eine Notfallversorgung hinausgeht und durch Therapieunterbrechungen und fehlende Kontinuität geprägt ist. Zivilgesellschaftliche Anlaufstellen und anonyme Behandlungsscheine kompensieren bestehende Lücken teilweise, bieten jedoch keine adäquate Alternative zur Regelversorgung.

**Diskussion:**

Menschen ohne Papiere sind trotz zivilgesellschaftlicher Initiativen unterversorgt, das Recht auf Gesundheit wird nicht umgesetzt. Zukünftige Forschungsarbeiten sind an den forschungsethischen Grundsätzen der Fürsorge, der Schadensminimierung und der Selbstbestimmung der Betroffenen auszurichten.

## Abstract (en)

**Background:**

Undocumented migrants in Germany face significant challenges in accessing healthcare. Although they are entitled to limited health services under the Asylum Seekers’ Benefits Act, they cannot utilize these services without risking detection and deportation due to reporting obligations of social authorities. This study systematically reviews the existing research on healthcare access in this population in Germany for the first time.

**Methods:**

The literature research was conducted following the PRISMA-ScR standard in the databases Medline, CINAHL, PSYNDEX, SocINDEX, and Juris, with additional information gathered through supplementary email inquiries and manual searches. Studies from the years 2005–2024 were included. The data were analyzed regarding legal, structural, and practical access barriers and treatment gaps as well as recommended measures.

**Results:**

A total of 88 studies were included, most of which are based on quantitative data collection. The studies reveal that access to healthcare for people without valid residence permits is usually limited to emergency care and is characterized by treatment interruptions and a lack of continuity in care. Humanitarian healthcare provision and anonymous healthcare vouchers partially compensate for existing gaps, but do not offer an adequate alternative to regular care.

**Discussion:**

Despite civil society initiatives, undocumented migrants are underserved, and the right to health is not implemented. Future research should be aligned with the research ethics principles of care, harm minimization, and the self-determination of those affected.

## Hintergrund

Menschen ohne gültige Aufenthaltspapiere^1^ haben in Deutschland einen erschwerten Zugang zur Gesundheitsversorgung. Sie sind mit einer konfliktreichen und widersprüchlichen Gesetzeslage konfrontiert, die dazu führt, dass sie den Kontakt mit dem Gesundheitswesen möglichst vermeiden oder hinauszögern: Grundsätzlich haben Menschen ohne Papiere Anspruch auf eingeschränkte Gesundheitsleistungen nach dem Asylbewerberleistungsgesetz. Vor der Inanspruchnahme von Gesundheitsleistungen müssen sie jedoch beim zuständigen Sozialamt einen Behandlungsschein beantragen. Dieses ist aufgrund der in Deutschland geltenden Übermittlungspflichten für öffentliche Stellen (§ 87 Abs. 2 Aufenthaltsgesetz (AufenthG)) gesetzlich verpflichtet, Personen, die keinen gültigen Aufenthaltsstatus haben, an die Ausländerbehörde zu melden. Menschen ohne Papiere können ihr Recht auf gesundheitliche Versorgung daher nicht in Anspruch nehmen, ohne Aufdeckung und Abschiebung zu riskieren. Ohne vorherigen Behördenkontakt können sie nur eine Notfallversorgung im Krankenhaus in Anspruch nehmen. Krankenhäuser sind im medizinischen Notfall verpflichtet, Hilfe zu leisten, auch wenn kein Krankenversicherungsschutz besteht. Die ärztliche Schweigepflicht verlängert sich in diesem Fall auf das Verwaltungspersonal des Krankenhauses und bis in die Sozialbehörde hinein, sodass eine Datenübermittlung an die Ausländerbehörde unzulässig ist. In der Praxis bestehen jedoch auch hier Unwissen und Unsicherheiten fort und es hängt vom Einsatz und Engagement der Häuser und Mitarbeitenden ab, ob angemessen versorgt wird [1–3]. Vielerorts versuchen niedrigschwellige medizinische Anlaufstellen für Menschen ohne Krankenversicherungsschutz, den fehlenden Zugang zur Regelversorgung zu kompensieren.

Mit der vorliegenden Arbeit wird der Forschungsstand zur gesundheitlichen Versorgung von Menschen ohne gültige Aufenthaltspapiere in Deutschland erstmals systematisch aufgearbeitet. Schwerpunkt lag dabei auf der Frage, welche rechtlichen und praktischen Zugangsbarrieren zur gesundheitlichen Versorgung für diese Personengruppen bestehen, wie die Versorgung derzeit ausgestaltet ist und welche rechtlichen und praktischen Maßnahmen zu ergreifen sind, um bestehende Versorgungslücken zu schließen.

Die Arbeit basiert konzeptionell auf einem menschenrechtlichen Verständnis von Gesundheit, das den diskriminierungsfreien Zugang zu Gesundheitsleistungen als universelles Recht betont [4]. Ergänzend dient das WHO-Modell der sozialen Determinanten von Gesundheit als analytischer Rahmen, um strukturelle und gesellschaftliche Ursachen gesundheitlicher Ungleichheit von Menschen ohne Papiere zu erfassen [5, 6].

## Methoden

Scoping-Reviews sind geeignet, um den Umfang, die Vielfalt und Charakteristika von Literatur zu einem wenig beforschten Thema darzustellen und Forschungslücken aufzuzeigen [7, 8]. Die Beschreibung des Vorgehens, der Datenextraktion, Datensynthese und Auswertung folgen den Preferred Reporting Items for Systematic Reviews and Meta-Analyses Extension for Scoping Reviews (PRISMA-ScR; [8]). Das a priori festgelegte Studienprotokoll wurde am 02.02.2024 auf der Seite des Open Science Framework registriert und am 05.03.2024 veröffentlicht (https://osf.io/u3nwk).

Die Ein- und Ausschlusskriterien (Tab. 1) orientierten sich an den Merkmalen „Population“, „Concept“ und „Context“ (PCC) sowie an den „Arten der Quellen“ nach Peters et al. [9].

- *Population*: Menschen ohne gültige Aufenthaltspapiere
- *Concept*: Gesundheitliche Versorgung (ambulant, stationär, rechtliche Situation, Behandlungsanlässe, wirtschaftliche Aspekte, Parallelstrukturen)
- *Context*: Situation in Deutschland, auch im Vergleich mit anderen Ländern
- *Arten der Quellen*: wissenschaftliche, empirische und fachpolitische Literatur; journalistische Artikel waren nicht Gegenstand der Analyse

**Tab. 1:** Ein- und Ausschlusskriterien des Scoping-Reviews

Kriterium	PCC-Merkmal	Einschlusskriterien	Ausschlusskriterien
*Menschen ohne gültige Aufenthaltspapiere*	„Population“	Die Literatur bezieht sich auf Menschen ohne gültige Aufenthaltspapiere und auf Personengruppen, denen Menschen ohne gültige Aufenthaltspapiere angehören können	Literatur bezieht sich auf keine der genannten Gruppen
			Im Volltext wird erkennbar, dass der Aufenthaltsstatus der untersuchten Population nicht Gegenstand der Untersuchung oder der Ergebnisse ist
*Gesundheitsversorgung*	„Concept“	Die Literatur beschäftigt sich mit den o. g. Gruppen im Kontext von Gesundheitsversorgung	Ein Zusammenhang mit Gesundheitsversorgung wird nicht beschrieben oder diskutiert
*Bezug zu Deutschland*	„Context“	Literatur zur Situation in Deutschland, auch im Vergleich mit anderen Ländern Oder Literatur, die deutsche Städte, Ansätze oder Anlaufstellen vergleicht	Literatur befasst sich nicht mit der Situation in Deutschland bzw. beinhaltet keinen Vergleich mit Deutschland oder deutschen Städten, Ansätzen oder Anlaufstellen
*Arten der Quellen*	–	Wissenschaftliche, empirische und fachpolitische Literatur aus den Bereichen Gesundheit, Soziales und Recht	Zeitschriftenartikel journalistischer Art, Handreichungen, politische Handlungsempfehlungen, Pressemitteilungen und -berichte, Ratgeber
		Graue Literatur (unveröffentlichte Qualifizierungsarbeiten ab Masterniveau, Evaluationsberichte, Statistiken)	
		Literatur in deutscher oder englischer Sprache	
		Veröffentlicht oder verfasst zwischen 2005 und 2024	

Der Suchzeitraum wurde auf die Jahre 2005–2024 eingegrenzt, da das Thema erst seit etwa 2005 mit dem Aufkommen zivilgesellschaftlicher medizinischer Anlaufstellen und fachlichem Engagement aus dem Gesundheitswesen verstärkt fachpolitische und wissenschaftliche Aufmerksamkeit erhalten hat.

Das Thema stellt einen Querschnittsbereich aus (Sozial‑)Medizin, Public Health und Versorgungsforschung sowie Rechtswissenschaften dar. Daher wurden Literaturdatenbanken aus all diesen Bereichen einbezogen (Tab. 2).

**Tab. 2:** Auswahl der Literaturdatenbanken nach Fachbereich

Bereich	Datenbank	Plattform
*Medizin/Versorgung*	Medline	Ovid
*Pflege*	CINAHL	EBSCOhost
*Psychologie*	PSYNDEX	EBSCOhost
*Sozialwissenschaften*	SocINDEX	EBSCOhost
*Rechtswissenschaften*	Juris	Juris

Um zusätzlich graue Literatur, wie zum Beispiel Evaluationsberichte oder zivilgesellschaftliche Arbeitspapiere, berücksichtigen zu können, wurden Sachverständigennetzwerke (u. a. wissenschaftliche Fachgesellschaften, fachpolitische Verbände und Nichtregierungsorganisationen – NGOs) per E‑Mail kontaktiert sowie *Google Scholar* systematisch nach relevanten Ergebnissen durchsucht. Mit dem Programm *Publish or Perish* wurde eine auf *Google Scholar* angepasste Suchstrategie angewendet und die ersten 300 Ergebnisse in das Screening eingeschlossen. So wurde sichergestellt, dass auch Publikationen, die nicht in den genannten Datenbanken auffindbar sind, eingeschlossen werden können.

Die Begriffe und Synonyme der Suchstrategie wurden mit Unterstützung eines thematisch einschlägigen Fachnetzwerks sowie bibliotheksmethodischer Beratung entwickelt. Die Suchstrategie wurde von einem zweiten Reviewer anhand der PRESS-Checkliste (Peer Review of Electronic Search Strategies; [10]) geprüft und entsprechend überarbeitet.

**Abb. 1:**
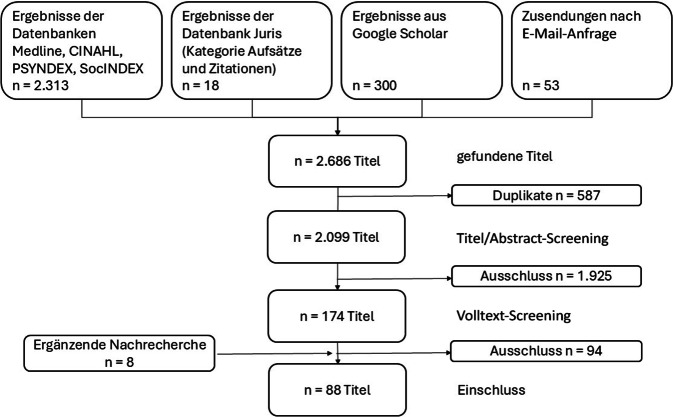
Literaturscreeningprozess. PRISMA-Flussdiagramm des Scoping-Reviews

Die identifizierten Quellen durchliefen einen mehrstufigen Screeningprozess (Abb. 1), bei dem zunächst die Duplikate entfernt wurden. Das Literaturscreening erfolgte mithilfe einer aufgrund der Ein- und Ausschlusskriterien festgelegten Checkliste: Titel/Abstract verblindet durch 2 Reviewer, anschließend Diskussion von Konflikten, zuletzt Screening der Volltexte durch eine Reviewerin. Titel ohne Abstract wurden nach spezifischen Schlüsselwörtern gescreent und gegebenenfalls ausgeschlossen. Der bis hierher beschriebene Screeningprozess fand im Rahmen der Masterarbeit von C. Ochs statt. Für den vorliegenden Artikel fanden Nachrecherchen statt, wodurch der Literaturkorpus leicht modifiziert und um 8 Quellen ergänzt wurde.

Die Ergebnisse wurden im Sinne einer narrativen Synthese aufbereitet, indem Studien nach zentralen Themen, Konzepten und theoretischen Bezügen geordnet und deskriptiv zusammengefasst wurden. Die Befunde wurden thematisch entsprechend den Fragestellungen zusammengestellt, um Gemeinsamkeiten, Unterschiede und Forschungslücken sichtbar zu machen [8, 9].

## Ergebnisse

### Merkmale der analysierten Literatur

Insgesamt wurden 88 Arbeiten in den Scoping-Review eingeschlossen, überwiegend wissenschaftliche Fachartikel (*n* = 44), daneben aber auch Forschungsberichte und graue Literatur (*n* = 30), Qualifizierungsarbeiten (*n* = 9) und Bücher bzw. Buchbeiträge (*n* = 5).

Der Mehrzahl der eingeschlossenen Arbeiten (*n* = 57) liegen empirische Daten zugrunde. In 31 Arbeiten werden quantitative Daten berichtet, wobei teils auf statistische Routinedaten aus der Beratungs- oder Versorgungspraxis von Anlaufstellen für Menschen ohne Krankenversicherungsschutz zurückgegriffen wird. In 19 Arbeiten werden qualitative Daten berichtet, wobei zumeist Expert:innen und Gesundheitsfachkräfte, teils auch Patient:innen interviewt wurden. In 7 Arbeiten wird ein Methoden-Mix angewendet. 31 nichtempirische Arbeiten wurden einbezogen, darunter 27 rechts-, sozial- oder politikwissenschaftliche Arbeiten und 4 systematische Literaturübersichten.

17 der eingeschlossenen Arbeiten befassen sich mit internationalen Vergleichen. 26 Arbeiten befassen sich mit der gesundheitlichen Versorgung von verschiedenen Personengruppen in vulnerablen Lebenssituationen und fokussieren nicht ausschließlich auf Menschen ohne gültige Aufenthaltspapiere. 27 Arbeiten beschreiben die Arbeit bestehender Anlaufstellen für Menschen ohne Krankenversicherungsschutz, darunter sind 4 wissenschaftliche Evaluationen. 4 Arbeiten befassen sich vorrangig mit der stationären Notfallversorgung von Menschen ohne gültige Aufenthaltspapiere bzw. ohne Krankenversicherungsschutz.

17 Arbeiten befassen sich mit bestimmten Versorgungsbereichen, wie zum Beispiel mit reproduktiver Gesundheit [11–15], Infektionsmedizin [16–20], Kinder und Jugendmedizin [21, 22], psychischer Gesundheit [23, 24], Palliativversorgung [25] oder Suchtmedizin [26] und Zahnmedizin [27].

### Rechtliche Barrieren und Versorgungslücken

#### Situation in Deutschland.

Die untersuchten Forschungsarbeiten zeigen übereinstimmend, dass Menschen ohne gültige Aufenthaltspapiere in Deutschland einen stark erschwerten Zugang zur Gesundheitsversorgung haben. Der eingangs skizzierte Sachverhalt, dass diese Menschen in der Praxis ihr Recht auf gesundheitliche Versorgung nicht in Anspruch nehmen können, wird in deutlichem Kontrast zu internationalen Vorgaben zum Recht auf Gesundheit gesehen [1–3, 28, 29].

Verschiedenste rechts- und politikwissenschaftliche Arbeiten [1, 3, 14, 28, 30] stellen heraus, dass die Übermittlungspflichten öffentlicher Stellen nach § 87 Abs. 2 AufenthG, denen die Sozialämter unterliegen, nicht mit dem Grundgesetz vereinbar sind sowie mit völkerrechtlichen und europarechtlichen Vorgaben zum Recht auf Gesundheit, wie dem Internationalen Pakt über wirtschaftliche, soziale und kulturelle Rechte (UN-Sozialpakt), dem UN-Übereinkommen zur Beseitigung jeder Form von Diskriminierung der Frau (CEDAW), dem Internationalen Übereinkommen zur Beseitigung jeder Form von rassistischer Diskriminierung (ICERD) und der UN-Kinderrechtskonvention (UN-KRK). Der deutsche Staat ist von UN-Menschenrechtsausschüssen wiederholt aufgefordert worden, die Übermittlungspflichten abzuschaffen, damit die betroffenen Personengruppen gesundheitliche Versorgung in Anspruch nehmen können.

Die Frage, ob Gesundheitsfachkräfte sich strafbar machen können, wenn sie Menschen ohne Papiere medizinische Hilfe leisten, ist rechtlich eindeutig geklärt. Das Bundesministerium des Innern stellte im Jahr 2007 klar, dass die gesundheitliche Versorgung von Menschen ohne Papiere nicht vom Tatbestand des § 96 Abs. 1 Nr. 2 AufenthG, Hilfe zum illegalen Aufenthalt, erfasst wird [31]. Im Jahr 2009 wurde dies durch die Allgemeine Verwaltungsvorschrift der Bundesregierung zum Aufenthaltsgesetz (AufenthG-VwV, Nr. 95.1.4) bekräftigt^2^ [33].

Ebenso ist eindeutig geklärt, dass die Übermittlungspflichten nicht für Berufsgruppen gelten, die gemäß § 203 Abs. 1 Strafgesetzbuch (StGB) der Schweigepflicht unterliegen, wie zum Beispiel Ärzt:innen, Psychotherapeut:innen sowie weitere Heilberufe, Sozialarbeiter:innen und Mitarbeitende in Familien‑, Sucht- und Schwangerschaftskonfliktberatungsstellen [32].

In Bezug auf die Notfallversorgung im Krankenhaus macht die Literatur deutlich, dass Krankenhäuser im medizinischen Notfall verpflichtet sind, Hilfe zu leisten, auch wenn kein Krankenversicherungsschutz besteht. Sie können nach § 6a AsylbLG, dem sogenannten „Nothelferparagraphen“, rückwirkend eine Kostenübernahme beim Sozialamt beantragen [2, 29].

Im Jahr 2009 stellt die Allgemeine Verwaltungsvorschrift der Bundesregierung zum Aufenthaltsgesetz (AufenthG-VwV, Nr. 88.2.4.0; [33]) klar, dass sich die ärztliche Schweigepflicht auch auf das mit der Abrechnung befasste Verwaltungspersonal eines Krankenhauses bezieht und sich bis in die Sozialbehörde hinein verlängert. Eine Datenübermittlung an die Ausländerbehörde ist in diesem Falle also unzulässig [2, 29, 33].

Einige Forschungsarbeiten untersuchen die Auswirkungen der bestehenden Ausschlüsse und zeigen, dass Menschen ohne gültige Aufenthaltspapiere oft über lange Zeit unversorgt bleiben und häufig an Komplikationen und Folgeerkrankungen leiden [34, 35]. Zudem führt Krankheit nicht selten zum Verlust der Lohnarbeit und zu ökonomischen Einbußen [36]. Von allen Personengruppen, die in Deutschland trotz der seit dem Jahr 2009 geltenden allgemeinen Krankenversicherungspflicht keinen oder nur eingeschränkten Zugang zur Regelversorgung haben, haben Menschen ohne Papiere die schlechtesten Zugangsmöglichkeiten zu gesundheitlicher Versorgung [37, 38].

Gottlieb und Ben Mocha (2018) beobachteten, dass die aus dem Menschenrecht auf Gesundheit abgeleitete Argumentation im politischen Diskurs zunehmend keine Wirkung mehr zeigt. So werde verstärkt argumentiert, dass Menschen ohne Papiere nicht Teil der Solidargemeinschaft seien [39].

#### Europäischer Vergleich.

Die Quellen zeigen, dass auch in anderen europäischen Ländern Menschen ohne gültige Aufenthaltspapiere einen erschwerten Zugang zu gesundheitlicher Versorgung haben. Zugangsbarrieren sind beispielsweise hohe Kosten, Sorgen vor Aufdeckung und Abschiebung sowie Unsicherheiten, fehlende Kenntnisse und restriktive Auslegungen der bestehenden Regelungen durch Mitarbeitende im Gesundheitswesen [40]. Als schwierig gilt insbesondere der Zugang zur ambulanten Regelversorgung und zu psychotherapeutischer Unterstützung. In mehreren Staaten der Europäischen Union (EU) ist auch der Zugang zur Notfallversorgung nicht gegeben [30]. Die Folgen sind eine verspätete Inanspruchnahme von Gesundheitsleistungen, fehlende Versorgungskontinuität und Unterversorgung [15, 24, 30, 40–43].

### Praktische Barrieren und Versorgungslücken

#### Sprachbarrieren.

In der Literatur werden Sprachbarrieren als maßgebliche Hürde beschrieben [24, 41]. Daten aus Anlaufstellen zeigen, dass zwischen 73–80 % der versorgten Menschen eine Sprachmittlung in der Versorgungssituation benötigen [44–50].

#### Unsicherheiten und fehlendes Wissen.

Unsicherheiten von Gesundheitsfachkräften und Verwaltungspersonal über die rechtliche Situation und die bestehenden Handlungsoptionen stellen einen relevanten limitierenden Faktor dar. Oft sind Mitarbeitenden in Gesundheitseinrichtungen die bestehenden Rechtsvorschriften nicht oder nicht ausreichend bekannt, was dazu führen kann, dass papierlose Patient:innen rechtswidrig an die Ausländerbehörde oder Polizei gemeldet werden [18, 30, 51]. Einige Arbeiten weisen darauf hin, dass Menschen ohne Papiere sich im Gesundheitswesen oftmals nicht gut auskennen und sie mit bestehenden Unterstützungsstrukturen nicht vertraut sind [51, 52]. Dagegen betont Huschke, dass Betroffene nicht selten über spezifische Kenntnisse zu informellen Netzwerken und Anlaufstellen verfügen und es nicht hilfreich sei, sie lediglich als hilflose Opfer darzustellen [53].

#### Fehlendes Vertrauen und rassistische Diskriminierung.

Als eine weitere Zugangsbarriere zu gesundheitlicher Versorgung wird fehlendes Vertrauen in das Gesundheitswesen und in staatliche Stellen angeführt. Misstrauen werde durch Berichte von Bekannten und durch Erfahrungen mit rassistischer Diskriminierung geschürt [12]. Das Vertrauen gegenüber Leistungserbringern der Regelversorgung scheint geringer zu sein als jenes gegenüber Anlaufstellen von NGOs [24].

### Ausgestaltung der Versorgung

#### Notfallversorgung durch Krankenhäuser.

Einige Forschungsarbeiten befassen sich mit der Notfallversorgung von Menschen ohne Krankenversicherungsschutz durch Krankenhäuser. Sie zeigen, dass Krankenhäuser ihre Kosten für die Behandlung selten oder nie erstattet bekommen [2, 54, 55]. Viele Krankenhäuser verlangen, dass Hilfesuchende die Kosten für eine Notfallbehandlung selbst tragen bzw. eine pauschale Summe zahlen, bevor sie ärztlich gesehen werden [55, 56]. Auch der verlängerte Geheimnisschutz scheint oft nicht gewährleistet zu sein, bisweilen informieren Mitarbeitende die Polizei, wenn Personen sich nicht ausweisen können und keinen Krankenversicherungsschutz nachweisen [2, 55]. Zudem kann das Fehlen von Leitfäden zur Versorgung von Menschen ohne Krankenversicherungsschutz dazu führen, dass eine Behandlung verweigert wird [24, 43]. Es konnte jedoch gezeigt werden, dass durch die Beratung von ausreichend qualifizierten Sozialdiensten in vielen Fällen ein Kostenträger für die Notfallversorgung ausfindig gemacht werden kann [37].

#### Parallelstrukturen der Versorgung.

In einigen Forschungsarbeiten stehen niedrigschwellige Anlaufstellen für Menschen ohne Krankenversicherungsschutz im Mittelpunkt der Analyse Diese Stellen gibt es in vielen Städten und einigen Bundesländern, sie unterscheiden sich stark in ihrer Ausgestaltung, Finanzierung und ihren Handlungsmöglichkeiten. Manche Anlaufstellen bieten eine sozialrechtliche Beratung an, einige verfügen über Behandlungsbudgets, über die sie eine eingeschränkte Versorgung im Regelsystem finanzieren können, manche Anlaufstellen vermitteln an Netzwerke ehrenamtlich unterstützender Ärzt:innen und andere halten selbst medizinische Sprechstunden vor [21, 38, 56, 57].

In den 1990er/2000er-Jahren haben vor allem kirchlich-karitative und politisch-aktivistische Initiativen praktische Unterstützungsarbeit geleistet. Diese haben unentgeltlich gearbeitet oder waren über Spendengelder finanziert. Seit den 2010er-Jahren werden vermehrt anonyme Behandlungsschein-Projekte und Clearingstellen errichtet, die durch kommunale oder Landesmittel finanziert werden [58]. Auch beim Öffentlichen Gesundheitsdienst gibt es Anlaufstellen für Menschen, die keinen Zugang zur Regelversorgung haben [1, 11, 18, 20, 29].

In vielen Forschungsarbeiten werden die Behandlungsanlässe und Diagnosen von Patient:innen, die sich in Anlaufstellen vorstellen, untersucht. Belastbare Rückschlüsse auf die Krankheitslast in der Population, auf bestehende Behandlungsbedarfe oder auf Unterschiede zwischen Menschen mit und ohne Krankenversicherungsschutz können aus den vorliegenden Daten jedoch nicht gezogen werden [23, 27, 35, 36, 44–50, 59–62].

### Grenzen von Parallelstrukturen und Einsatz für strukturelle Lösungen

In mehreren Forschungsarbeiten wird aufgezeigt, dass die zumeist prekär ausgestatteten Anlaufstellen der Parallelstrukturen die bestehenden Versorgungslücken nach wie vor nur unzureichend füllen. Dies reiche nicht aus, um die staatliche Pflicht zur Gewährleistung eines gesundheitlichen Existenzminimums zu erfüllen [3, 63]. Viele Arbeiten beschreiben zudem die praktischen Grenzen von Parallelstrukturen, wie geringe finanzielle und personelle Ressourcen [21, 27, 63], eingeschränkte Öffnungszeiten [21] und, insbesondere bei ehrenamtlich organisierten Anlaufstellen, eine oft große Fluktuation der Helfenden [54]. Bei kostenintensiven Behandlungen chronischer Erkrankungen stießen Anlaufstellen regelmäßig an ihre Grenzen [27, 34], bestehende Behandlungsbudgets seien oft nicht ausreichend [62]. Komplexere Versorgungsprozesse zu koordinieren und Krankenhausaufenthalte zu organisieren sei herausfordernd [34]. Für Ärzt:innen gehe eine über Parallelstrukturen organisierte Versorgung mit einem größeren Mehraufwand einher [38] und für Anlaufstellen werde es zunehmend schwieriger, kooperierende Gesundheitseinrichtungen zu finden [62].

Als problematisch werden zudem Machthierarchien und Abhängigkeitsstrukturen in Parallelstrukturen beschrieben [54]. Von Hilfesuchenden werde bisweilen erwartet, dass sie bedürftig erscheinen und Dankbarkeit gegenüber den ehrenamtlich Helfenden äußern [64]. Im öffentlichen Diskurs könne das Nebeneinander von Regelversorgung und Parallelstrukturen die Annahme befördern, dass manche Personengruppen eine gesundheitliche Versorgung in geringerem Maße verdient hätten und ihre Versorgung daher mit geringerem Aufwand geleistet werden könne [34, 56].

In Hinblick auf strukturelle Lösungen heben einige Arbeiten hervor, dass viele zivilgesellschaftliche Initiativen nicht nur praktische Unterstützungsarbeit leisten, sondern auch für politische Lösungen eintreten, beispielsweise durch die Mitwirkung in Bündnissen wie der Kampagne „GleichBeHandeln“ [56]. Oft wird betont, dass man langfristig auf die Abschaffung der nur als Interimslösung gedachten Anlaufstellen hinarbeite [38, 57, 62]. Zugleich wird in der Literatur deutlich, dass Politik und Verwaltung die Parallelstrukturen von Beginn an überwiegend wertschätzen und beispielsweise auf polizeiliche Kontrollen verzichteten [65, 66].

Kommunen wählen unterschiedliche Strategien und Praktiken, um Menschen ohne Papiere Zugang zu gesundheitlicher Versorgung zu ermöglichen, wie mehrere Arbeiten aufzeigten [67–69]. Diese beinhalten, die durch nationales Recht vorgegebenen rechtlichen Rahmenbedingungen breit zu interpretieren, Netzwerke aufzubauen, ressortübergreifende Ansätze zu entwickeln und eng mit zivilgesellschaftlichen Akteur:innen zusammenzuarbeiten [67, 68]. Benannt wurde auch die Möglichkeit, Zugang zu gesundheitlicher Versorgung über einen sog. Stadtausweis sicherzustellen [69].

## Diskussion

Der vorliegende Scoping-Review bietet eine systematische Aufarbeitung des Forschungsstandes zur gesundheitlichen Versorgung von Menschen ohne gültige Aufenthaltspapiere in Deutschland. Die Ergebnisse verdeutlichen, dass der Zugang zur Gesundheitsversorgung als intermediäre soziale Determinante von Gesundheit [2, 3] für Menschen ohne Papiere stark eingeschränkt ist. Sie gehören zu den am schlechtesten versorgten Gruppen im Gesundheitswesen, obwohl sie durchaus über Leistungsansprüche verfügen. Insbesondere die Übermittlungspflichten nach § 87 Abs. 2 AufenthG verhindern die Inanspruchnahme von Gesundheitsleistungen. Dies steht im Widerspruch zu internationalen menschenrechtlichen Verpflichtungen und lässt sich nicht mit der ethischen Norm des Rechts auf Gesundheit im Sinne einer angemessenen medizinischen Versorgung vereinbaren [4].

Hinzu kommen zahlreiche praktische Zugangshürden zu gesundheitlicher Versorgung, wie beispielsweise Sprachbarrieren. Unsicherheiten von Mitarbeitenden im Gesundheitswesen können dazu führen, dass aus Sorge, das Falsche zu tun, lieber gar nichts getan wird, dass Handlungsspielräume unterschätzt und notwendige Behandlungen unterlassen werden. Im schlimmsten Fall kann dies dazu führen, dass Gesundheitsfachkräfte die Daten rechtswidrig an Ausländerbehörden oder Polizei weitergeben, da sie fälschlich meinen, dazu verpflichtet zu sein.

In der Analyse der vorliegenden Forschungsarbeiten fiel auf, dass auch einige der neueren Arbeiten hier nicht für Klarheit sorgen, sondern bestehende Unsicherheiten noch verstärken. Einige Arbeiten deuten an, dass Gesundheitsfachkräfte bei der Versorgung von Menschen ohne gültige Aufenthaltspapiere in einem „rechtlichen Graubereich“ handeln würden. Andere Arbeiten implizieren, die in Deutschland bestehenden Übermittlungspflichten bezögen sich auf Gesundheitsfachkräfte. Derartige Ungenauigkeiten bergen die Gefahr, dass falsche Informationen weiterverbreitet und Unsicherheiten unter Gesundheitsfachkräften noch verstärkt werden. Es liegt in der Verantwortung von Forschenden, dem entschieden entgegenzuwirken.

Auch wenn es mittlerweile vielerorts Anlaufstellen für Menschen ohne Krankenversicherungsschutz gibt, können diese nur eine fragmentierte und unzureichende Gesundheitsversorgung bieten und eine bedarfsgerechte Regelversorgung nicht ersetzen. Zum einen spielen regionale Unterschiede eine Rolle: Solche Angebote sind vor allem in größeren Städten, jedoch kaum in ländlichen Räumen vorhanden. Zum anderen stoßen diese Strukturen insbesondere bei chronischen und schweren Erkrankungen, die aufwendige und kostenintensive Behandlungen erfordern, an ihre Grenzen. Viele Anlaufstellen weisen immer wieder deutlich auf die Probleme hin, die durch den Aufbau paralleler Versorgungsstrukturen geschaffen werden, und setzen sich für politische Lösungen ein, die einen Zugang zur gesundheitlichen Regelversorgung für alle Menschen sicherstellen.

Trotz der zunehmenden wissenschaftlichen Auseinandersetzung mit dem Thema bestehen weiterhin Forschungslücken: Es fehlen Studien zur Unter- und Fehlversorgung durch Parallelstrukturen, beispielsweise im Rahmen der Langzeitversorgung bei chronischen und Mehrfacherkrankungen. Der generell unzureichende Forschungsstand zu rassistischer Diskriminierung in der Gesundheitsversorgung in Deutschland zeigt sich auch in der Literatur zu diesem Thema [70]. Es fehlen zudem partizipative Forschungsansätze, die die Perspektiven und die Handlungsfähigkeit (Agency) der Betroffenen stärker einbeziehen [71, 72]. Im Sinne einer reflexiven wissenschaftlichen Professionalität wären ein stärkerer Einbezug von Betroffenenperspektiven und auch die jeweilige Selbstpositionierung der Forschenden wünschenswert [73]. Zudem sind weitere Personengruppen, wie beispielsweise viele in Deutschland lebende Bürger:innen anderer EU-Staaten, ebenfalls weitgehend vom Zugang zu Gesundheitsleistungen ausgeschlossen.^3^ Eine systematische Erfassung des Forschungsstands zu dieser Gruppe wäre ebenfalls sinnvoll, auch um Versorgungslücken im Vergleich besser identifizieren zu können.

### Limitationen.

Diese Studie unterliegt einigen methodischen Limitationen. Die Literaturrecherche wurde bewusst sehr breit angelegt, es wurde keine Einschränkung auf wissenschaftliche Fachartikel vorgenommen, sondern auch fachpolitische Arbeiten wurden berücksichtigt. Dies hat zur Folge, dass die Entscheidung über Ein- oder Ausschluss nicht immer eindeutig zu fällen war. Es ist nicht gänzlich auszuschließen, dass relevante Arbeiten übersehen wurden. Auch ein Auswahl- bzw. Interpretationsbias durch die Reviewer:innen ist nicht auszuschließen. Viele Arbeiten wurden von zivilgesellschaftlichen Initiativen und Organisationen durchgeführt. Diese bringen einerseits wertvolle Forschungsperspektiven mit, da sie über Wissen, Erfahrungen und Zugänge verfügen, zugleich ist aber in der Interpretation der Ergebnisse auch eventuelle Parteilichkeit in ihrer Forschung zu berücksichtigen. Schließlich weisen solche Studien teils ressourcenbedingt methodische Begrenzungen auf und beruhen zumeist auf kleineren regionalen Stichproben. Umfangreichere vergleichende Analysen, auch mit anderen europäischen Staaten, wären wünschenswert, auch um Transferpotenziale von Good-Practice-Modellen für eine bedarfsgerechte Versorgung zu identifizieren.

## Fazit

Menschen ohne gültige Aufenthaltspapiere in Deutschland sind erheblichen Zugangshürden zur Gesundheitsversorgung ausgesetzt und unterversorgt. Rechtliche und praktische Barrieren verschränken sich und verhindern eine adäquate Versorgung, selbst in Notfällen. Zivilgesellschaftliche Parallelstrukturen spielen eine essenzielle Rolle, sind aber keine bedarfsgerechte Alternative zur Regelversorgung. Zukünftige Forschung sollte sich angesichts des sensiblen Handlungsfeldes explizit forschungsethisch positionieren und verstärkt Betroffenenperspektiven einbeziehen.

## Footnotes

1 Im Folgenden wird die Bezeichnung „Menschen ohne gültige Aufenthaltspapiere“ bzw. verkürzt „Menschen ohne Papiere“ für Personen verwendet, die sich ohne asyl- oder ausländerrechtlichen Aufenthaltstitel, ohne Duldung und ohne behördliche Erfassung in Deutschland aufhalten bzw. ohne Arbeitserlaubnis arbeiten. Die Vielfalt der Begrifflichkeiten sowie der damit einhergehende kritische Diskurs zu Konnotationen und Diskriminierungsrisiken können im Artikel nicht abgebildet werden; auf die Bedeutung der Wortwahl soll hier jedoch hingewiesen werden. Zusätzlich wird der Begriff „Menschen ohne Krankenversicherungsschutz“ verwendet, der auch weitere Personengruppen (z. B. Bürger:innen anderer EU-Staaten und deutsche Staatsangehörige ohne Krankenversicherung) mit vergleichbaren Zugangsbarrieren zum Gesundheitssystem umfasst.

2 „Handlungen von Personen, die im Rahmen ihres Berufes oder ihres sozial anerkannten Ehrenamtes tätig werden (insbesondere Apotheker, Ärzte, Hebammen, Angehörige von Pflegeberufen, Psychiater, Seelsorger, Lehrer, Sozialarbeiter, Richter oder Rechtsanwälte), werden regelmäßig keine Beteiligung leisten, soweit die Handlungen sich objektiv auf die Erfüllung ihrer rechtlich festgelegten bzw. anerkannten berufs-/ehrenamtsspezifischen Pflichten beschränken“ [33].

3 Seit Verabschiedung des Leistungsausschlussgesetzes (§ 7 SGB II, § 23 SGB XII) sind erwerbslose Bürger:innen anderer EU-Staaten, wenn sie länger als 3 Monate und weniger als 5 Jahre in Deutschland leben, von Sozial- und Gesundheitsleistungen weitgehend ausgeschlossen. Innerhalb von 2 Jahren können sie für maximal einen Monat Überbrückungsleistungen erhalten, die auch eingeschränkte Gesundheitsleistungen beinhalten.
